# Supplementary material for: The Systems Biology Research Tool: evolvable open-source software
Source: BMC Syst Biol. 2008 Jun 29;2:55. doi: 10.1186/1752-0509-2-55 (PMC2446383; doi:10.1186/1752-0509-2-55)
Supplement: Additional file 1 — SBRT Archive. An archive of the current version of the Systems Biology Research Tool. [file 1752-0509-2-55-S1.zip › sbrt-1.4.0/doc/developers_guide/api/sbrt/ext/mma/algebra/MathematicaSolver.html]

MathematicaSolver


|  |  |  |
| --- | --- | --- |
| **Overview** \| **Package** \| **Class** \| **Use** \| **Tree** \| **Deprecated** \| **Index** \| **Help** | |  |
| PREV CLASS   **NEXT CLASS** | **FRAMES**    **NO FRAMES**     **All Classes** |  |
| SUMMARY: NESTED \| FIELD \| CONSTR \| METHOD | DETAIL: FIELD \| CONSTR \| METHOD |  |


---


## sbrt.ext.mma.algebra Class MathematicaSolver<V>

```
java.lang.Object
  sbrt.ext.mma.algebra.MathematicaSolver<V>
```

**Type Parameters:**: `V` - the variable type.

**All Implemented Interfaces:**: LinearSystemSolver<V>

---

``` public class MathematicaSolver<V> extends java.lang.Object implements LinearSystemSolver<V> ```

This class is used to solve systems of linear equations using Mathematica.

**Author:**
:   This class was written and documented by Jeremiah Wright while in the
    Wagner lab.

---

**Constructor Summary**

`MathematicaSolver(java.lang.String kernelLinkCmd)`
  
          Creates a new linear system solver.

`MathematicaSolver(java.lang.String[] kernelLinkArgs)`
  
          Creates a new linear system solver.


| **Method Summary** | |
| --- | --- |
| `void` | `close()`             Closes the link to the Mathematica kernel. |
| `protected  void` | `finalize()`             Calls `close()`. |
| `static void` | `main(java.lang.String[] args)`             Used for testing purposes. |
| `LinearSystemSolution<V>` | `solve(java.util.Map<LinearComb<V>,java.lang.Double> equations)`             Computes and returns a solution to the provided system of linear equations. |

**Methods inherited from class java.lang.Object**

`clone, equals, getClass, hashCode, notify, notifyAll, toString, wait, wait, wait`

**Constructor Detail**

### MathematicaSolver

```
public MathematicaSolver(java.lang.String[] kernelLinkArgs)
```

:   Creates a new linear system solver.

    **Parameters:**: `kernelLinkArgs` - the argument for the Mathematica JLink method `MathLinkFactory.createKernelLink(String[])`. **Throws:**: `ExternalSoftwareException` - if a `MathLinkException` is thrown.

---


### MathematicaSolver

```
public MathematicaSolver(java.lang.String kernelLinkCmd)
```

:   Creates a new linear system solver.

    **Parameters:**: `kernelLinkCmd` - the argument for the Mathematica JLink method `MathLinkFactory.createKernelLink(String)`. **Throws:**: `MathematicaException` - if a `MathLinkException` is thrown.


**Method Detail**

### main

```
public static void main(java.lang.String[] args)
```

:   Used for testing purposes.

    :   **Parameters:**: `args` - ignored.

---


### close

```
public void close()
```

:   Closes the link to the Mathematica kernel.

---


### finalize

```
protected void finalize()
                 throws java.lang.Throwable
```

:   Calls `close()`.

    :   **Overrides:**: `finalize` in class `java.lang.Object`
    :   **Throws:**: `java.lang.Throwable`

---


### solve

```
public LinearSystemSolution<V> solve(java.util.Map<LinearComb<V>,java.lang.Double> equations)
```

:   Computes and returns a solution to the provided system of linear
    equations.

    :   **Specified by:**: `solve` in interface `LinearSystemSolver<V>`
    :   **Parameters:**: `equations` - linear combinations each mapped to a `Double`. Each key-value pair represents the left and right hand sides, respectively, of an equation. **Returns:**: a solution to the provided system of linear equations. **Throws:**: `MathematicaException` - if a `MathLinkException` is thrown.: `java.lang.IllegalStateException` - if `close()` has already been called.


---


|  |  |  |
| --- | --- | --- |
| **Overview** \| **Package** \| **Class** \| **Use** \| **Tree** \| **Deprecated** \| **Index** \| **Help** | |  |
| PREV CLASS   **NEXT CLASS** | **FRAMES**    **NO FRAMES**     **All Classes** |  |
| SUMMARY: NESTED \| FIELD \| CONSTR \| METHOD | DETAIL: FIELD \| CONSTR \| METHOD |  |


---
